# Efficient Elimination of mtDNA from Mammalian Cells with 2′,3′-Dideoxycytidine

**DOI:** 10.3390/dna4030013

**Published:** 2024-07-04

**Authors:** Natalya Kozhukhar, Mikhail F. Alexeyev

**Affiliations:** Department of Physiology and Cell Biology, University of South Alabama, Mobile, AL 36688, USA;

**Keywords:** mtDNA depletion, rho-0 cells, ddC, HeLa, A549, HT1080

## Abstract

Mammalian cell lines devoid of mitochondrial DNA (mtDNA) are indispensable in studies aimed at elucidating the contribution of mtDNA to various cellular processes or interactions between nuclear and mitochondrial genomes. However, the repertoire of tools for generating such cells (also known as rho-0 or ρ^0^ cells) remains limited, and approaches remain time- and labor-intensive, ultimately limiting their availability. Ethidium bromide (EtBr), which is most commonly used to induce mtDNA loss in mammalian cells, is cytostatic and mutagenic as it affects both nuclear and mitochondrial genomes. Therefore, there is growing interest in new tools for generating ρ^0^ cell lines. Here, we examined the utility of 2′,3′-dideoxycytidine (ddC, zalcitabine) alone or in combination with EtBr for generating ρ^0^ cell lines of mouse and human origin as well as inducing the ρ^0^ state in mouse/human somatic cell hybrids. We report that ddC is superior to EtBr in both immortalized mouse fibroblasts and human 143B cells. Also, unlike EtBr, ddC exhibits no cytostatic effects at the highest concentration tested (200 μM), making it more suitable for general use. We conclude that ddC is a promising new tool for generating mammalian ρ^0^ cell lines.

## 1. Introduction

Mitochondrial DNA (mtDNA) plays a central role in the biology of mitochondria and, indeed, in all cellular processes, which are affected, either directly or indirectly, by its presence or absence. In cultured cells, knockouts (KOs) of genes essential for mtDNA replication result in the loss of mtDNA [1], whereas the whole-body KOs of the same genes in experimental animals are usually embryonically lethal [2–7]. To our knowledge, there are no reports of multicellular organisms retaining their viability after complete mtDNA loss. However, derivatives of cultured cells lacking mtDNA can be obtained. The resulting cells (called rho-0 (ρ^0^) cells) lack respiratory function and are auxotrophic for uridine and pyruvate [8–10].

ρ^0^ cells are useful as recipients in cybrid technology, which aims to study the effects of mtDNA mutations in a uniform genetic background [11–19]. Indeed, phenotypic manifestations of mtDNA mutation depend on complex and as yet incompletely understood interactions of nuclear and mitochondrial genomes. One prominent example of the importance of taking into account these interactions is the case of the G13997A mtDNA mutation in mouse cells. Initially, this mutation was described as promoting metastasis [20]. Transmitochondrial mice carrying this mutation had an increased incidence of lymphoma [21]. However, this increase was only observed in the B6 nuclear background, not in other mouse strains [22]. Therefore, ρ^0^ cells are an important tool in deciphering the physiological consequences of mtDNA mutations.

Historically, ρ^0^ cells were obtained by a variety of techniques, including treating cells with inhibitors of mtDNA replication, such as ethidium bromide (EtBr) [8,9,23–26] or ditercalinium [27–30], or by targeting enzymatic damage to mtDNA [31–34]. Each of these techniques has its limitation(s), and therefore, there is ongoing interest in new tools for mtDNA elimination from cultured cells.

Zalcitabine (2′,3′-dideoxycytidine, ddC) was initially developed as an antiretroviral drug to combat HIV infections. ddC is an analog of the cellular DNA precursor 2′-deoxycytidine-5′-triphosphate (dCTP) and is converted inside the cells into 2′,3′-dideoxycytidine-5′-triphosphate (ddCTP) regardless of their infection status [35]. ddCTP suppresses viral integration into the host genome by inhibiting nascent DNA chain elongation by viral reverse transcriptase due to the absence of a hydroxyl group at the 3′ position. Zalcitabine’s side effects, including peripheral neuropathy, stomatitis, and mouth ulcers, limit its dosage and have been linked to the inhibition of mitochondrial DNA polymerase gamma (POLG), which results in mtDNA depletion [36–41]. Other so-called “D-drugs” (didanosine (ddI) and stavudine (d4T)) also induce mtDNA depletion [36]. Perplexingly, “non-D-drugs” can also induce mtDNA depletion even though they do not inhibit POLG [37,38,42]. However, no complete loss of mtDNA was reported in patients undergoing antiretroviral therapy; the average mtDNA loss was only about 53% [36]. Therefore, whether zalcitabine or any other nucleoside reverse transcriptase inhibitors can induce complete mtDNA loss in cells in vivo or in culture remains unclear.

In this study, we examined the effects of chronic ddC treatment on mtDNA maintenance in various cell lines. We concluded that ddC alone, or in combination with EtBr, is an efficient means of inducing the ρ^0^ state, which is superior to that achieved by the broadly used EtBr.

## 2. Materials and Methods

### 2.1. Cell Lines and Culture Conditions

MEF#5 is a clone of spontaneously immortalized mouse embryonic fibroblasts with genotype TFAM^loxP/loxP^, Gt(ROSA)26^Sor+/lox-Stop-lox-mito-YFP^. Primary embryonic fibroblasts were kindly provided by Nils-Goran Larsson [43]. 143B, HepG2, and HeLa cells were from ATCC (CRL-8303, HB-8065, and CCL-2, respectively). HT1080 cells were from the University of California, Berkeley cell culture core. A549 cells were from the laboratory collection. JCRB2201, 2202, 2207, 2214, and 2222 and human/mouse somatic cell hybrids containing single human chromosomes (Chr1, Chr2, Chr7, Chr14, or Chr22, respectively) were from the cell line repository at the National Institutes of Biomedical Innovation, Health, and Nutrition, Japan and were obtained through Sekisui XenoTech, Kansas City, KS via an agreement with Tottori University [44]. The 143B#6 and 5460#5 cell lines have been described previously [1].

All cells were propagated in Dulbecco’s modified Eagle’s medium (DMEM) containing 4.5 g/L glucose, 10% fetal bovine serum, 50 μg/mL gentamycin, 50 μg/mL uridine, and 1 mM sodium pyruvate in a humidified atmosphere containing 5% CO_2_ at 37 °C. This modification of the medium (+UP) is permissive for the growth of cells devoid of mtDNA (ρ^0^ cells). For cultivation of JCRB cells, media were additionally supplemented with 1 mg/mL G418 (JCRB2201, 2202, and 2207) or 1 mg/mL hygromycin (JCRB2214 and 2222) to select for the presence of human chromosomes.

The identity of the cell lines used in this study was confirmed by STR analysis (LabCorp, Burlington, NC, USA). The cell lines were quarterly tested for mycoplasma contamination by PCR.

### 2.2. Determination of mtDNA Copy Number (mtCN) by Direct Digital Droplet PCR

mtCN was determined as described previously [45]. Briefly, cells were collected by trypsinization and counted, and ~10^6^-cell pellets were generated and frozen at −80 °C. Pellets were resuspended in PBS at ~10,000 cells/μL, and 10 μL aliquots were removed and mixed with 90 μL of solution containing 50 μg proteinase K, 40 μL of H_2_O, and 50 μL of the DirectPCR solution (Genprice Inc., San Jose, CA, USA, Cat# 388–302-C). The mix was incubated at 50 °C for 30 min and then at 95 °C for another 30 min. The solution was adjusted to 500 μL with H_2_O, and 3 μL of the resulting solution was used as a template in 20 μL ddPCR to determine nuclear DNA (nDNA) content using the primers and probes listed in Table 1. For mtDNA quantification, nDNA samples were diluted 500-fold, and 3 μL of the resulting dilution was used in 20 μL ddPCRs with the primers and probes listed in Table 1. dddPCRs contained 0.9 μM of each forward and reverse primer, 0.25 μM probe, 10 μL of the 2× ddPCR Supermix for Probes (No dUTP, Bio-Rad, Hercules, CA, USA Cat#1863023), 10 units of EcoRI HF restriction enzyme (New England Biolabs, Beverly, MA, USA, Cat# R3101S), and the balance of water. The cycling parameters were as follows: initial denaturation for 10 min at 95 °C, followed by 40 cycles of 20 s at 94 °C + 1 min at 60 °C, 10 min at 98 °C, and held at 4 °C. Each sample was measured in 2 technical replicates. To calculate mtCN per cell, the concentration of mtDNA targets was multiplied by the dilution factor and divided by the 0.5× concentration of nDNA targets. Each mtDNA template concentration was combined with each nDNA template concentration, generating four mtCN values for each sample.

### 2.3. Clonal Isolation of ρ^0^ Cells and Confirmation of the ρ^0^ Status

Upon mtCN in the treated cells dropping below 1, as judged by dddPCR, the cells were serially diluted, plated onto 150 mm tissue culture plates, and allowed to grow until the colonies reached 1–2 mm in diameter (2–3 weeks, for different cell lines). Using cloning disks, the smallest colonies were identified and picked for transfer into 24-well tissue culture plates. Upon expansion in DMEM+UP media containing appropriate antibiotics, cells were dissociated with 100 μL of trypsin, and 10 μL was removed for the detection of mtDNA using DirectPCR reagent as recommended by the manufacturer with primers listed in Table 1 [33].

## 3. Results

### 3.1. ddC Is More Effective than EtBr in mtDNA Elimination from Immortalized Mouse Embryonic Fibroblasts

**Figure 1.**
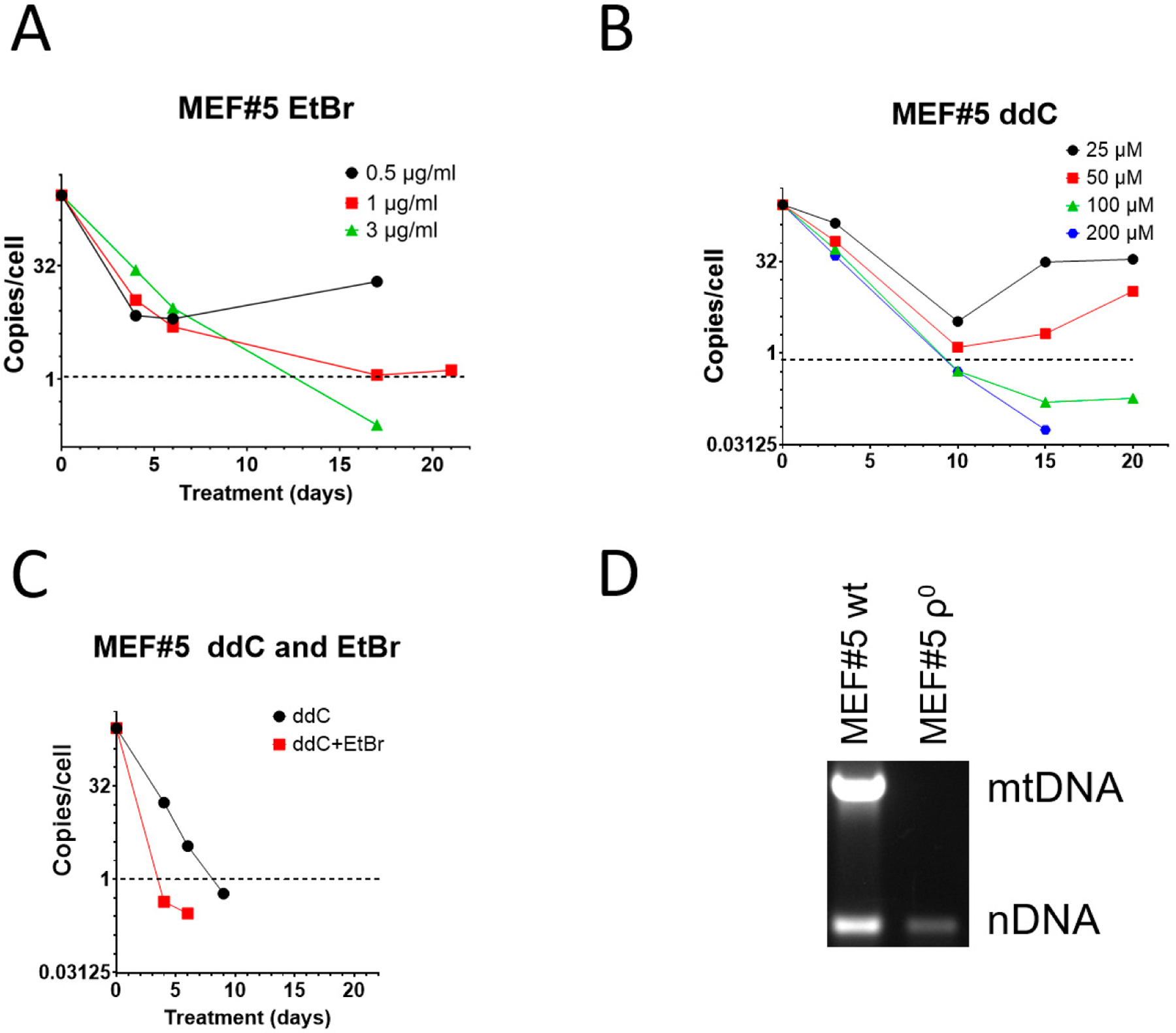
mtDNA depletion in MEF#5 with EtBr, ddC, and their combination. (**A**) Low concentrations of EtBr are ineffective in mtDNA depletion. (**B**) Low concentrations of ddC are ineffective in mouse cells, whereas higher concentrations are more effective than EtBr. (**C**) A combination of 200 μM ddC and 0.5 μg/mL EtBr is more effective than either drug alone. (**D**) MEF#5 cells, cloned after ddC treatment and expanded in the media without ddC, contain no mtDNA.

EtBr remains the most popular mtDNA elimination agent [8,9]. However, it has been suggested that in mouse cells, EtBr induces resistance, making this reagent unsuitable for the isolation of mouse ρ^0^ cell lines [27,28,46]. This assertion does not align with our previous experience with 3T3 cells, which were susceptible to EtBr treatment [47]. Nevertheless, 3T3 cells required higher EtBr concentrations to be effective. Therefore, we compared the effectiveness of EtBr and ddC for mtDNA elimination from spontaneously immortalized mouse embryonic fibroblasts MEF#5. Consistent with our previous observations, low concentrations (0.5 and 1 μg/mL) of EtBr were ineffective in MEF#5 cells and produced resistant cells. However, at 3 μg/mL, EtBr resulted in mtDNA depletion below an arbitrary threshold of an average of 1 mtDNA copy per cell in the population (Figure 1A). It was chosen because, at least in theory, at this threshold, more than 1/3 of cells in the population would be expected to contain no mtDNA at all, and ρ^0^ cells could be isolated by analyzing random clones from serially diluted cells. At 10 μg/mL EtBr, MEF#5 cells ceased to proliferate after several days in culture.

When compared to EtBr, zalcitabine was more effective in mtDNA depletion, and the 1 mtDNA copy per cell threshold was achieved in less than 10 days (compared to ~12 days with EtBr) at ddC concentrations of 100 and 200 μM (Figure 1B). Importantly, low concentrations of ddC were ineffective in MEF#5 cells and resulted in the growth of resistant cells (Figure 1B). This behavior is similar to that of low concentrations of EtBr (Figure 1A).

The effects of EtBr and ddC were additive. Treatment with a low EtBr concentration that induced resistance (0.5 μg/mL) with ddC at 200 μM was more efficient than treatment with ddC alone in that the threshold was reached in 4 days rather than 8 days (Figure 1C). At the same time, ddC did not exacerbate the cytostatic effects of EtBr. We believe this observation might help combat spontaneous resistance to each of these compounds used individually.

To confirm that ddC can be used to generate murine cell lines permanently depleted of mtDNA, we cloned MEF#5 after depletion with ddC and verified that the resulting cells, after expansion in media without ddC, contained no mtDNA (Figure 1D).

### 3.2. ddC Is More Effective than EtBr in mtDNA Elimination from Human Cells

**Figure 2.**
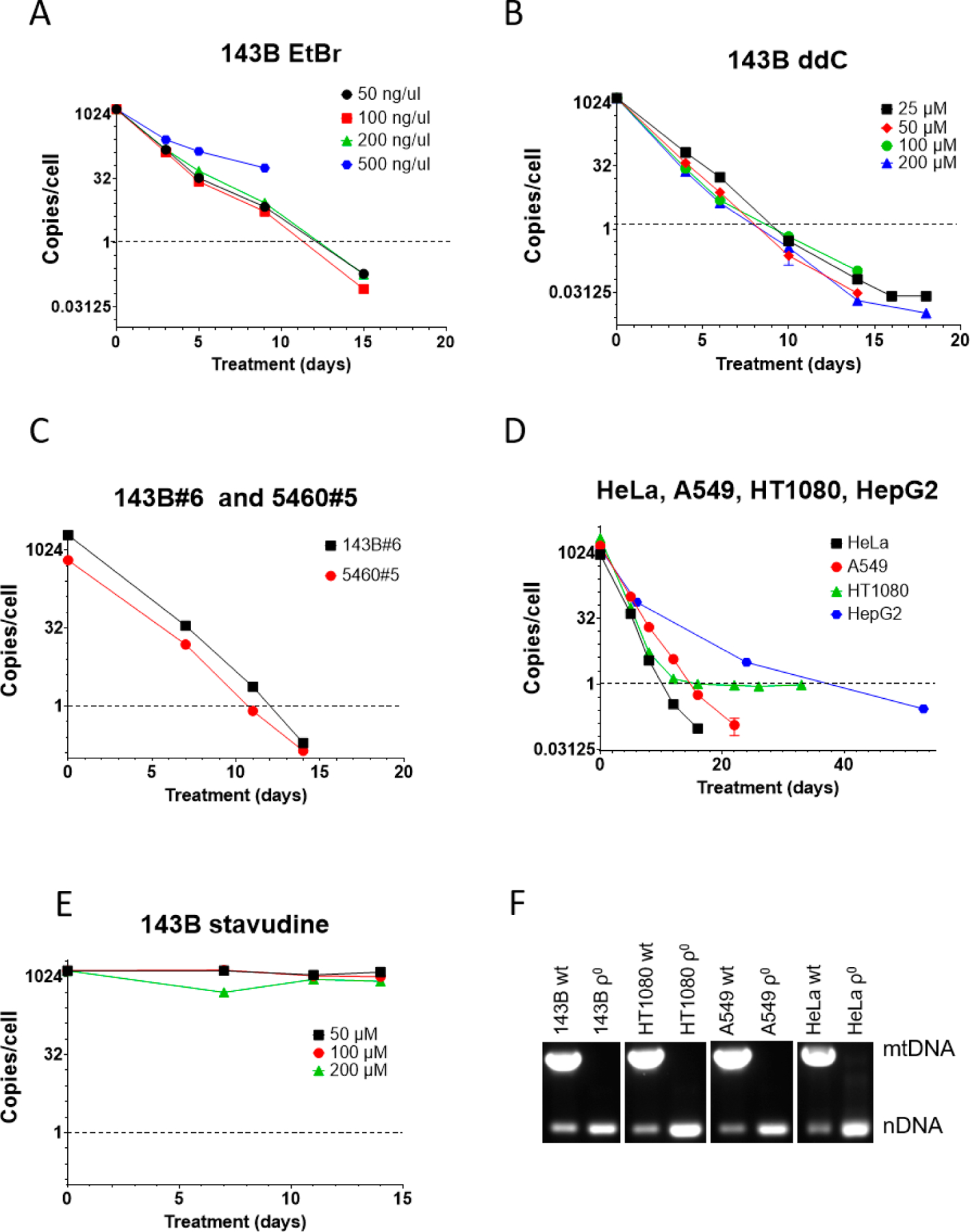
mtDNA depletion in human cells with EtBr and ddC. (**A**) High concentrations of EtBr are cytostatic and resulted in the termination of the experiment at 9 days, whereas lower concentrations of EtBr were equally effective. (**B**) ddC was almost equally effective at all tested concentrations. No cytostatic effect was observed, even at the highest tested concentration. (**C**) An amount of 200 μM ddC is effective for mtDNA depletion in GeneSwapped derivatives of 143B cells. (**D**) An amount of 200 μM ddC is effective for reducing mtCN below the 1 mtDNA copy per cell threshold in HeLa, A549, HT1080, and HepG2 cells. (**E**) Unlike ddC, stavudine (d4T) is ineffective for mtDNA depletion in 143B cells at all tested concentrations. (**F**) Analysis of mtDNA content in clonal cells isolated after ddC treatment by duplex PCR, with two pairs of primers specific for nuclear DNA and mtDNA.

We also compared ddC performance to that of EtBr in human osteosarcoma 143B cells. Here, too, a higher concentration of EtBr (500 ng/mL) was cytostatic and not only resulted in growth arrest and cell death but also in a slower mtDNA depletion, presumably because of the loss of the benefit of mtDNA dilution by cell division (Figure 2A). Importantly, cytostatic EtBr concentration in 143B cells was 20 times lower than in MEF#5 cells, suggesting greater sensitivity of human cells to EtBr as compared to mouse cells. This suggestion agrees with our observations in other cell lines. Other concentrations of EtBr (50, 100, and 200 ng/mL) were equally effective and reduced mtCN to an average 1 mtDNA copy per cell threshold in 12–13 days (Figure 2A).

In contrast to mouse MEF#5 cells, in 143B cells, ddC’s effectiveness was comparable at all concentrations tested, reducing mtCN to the threshold in 8–10 days, which is faster than with EtBr (Figure 2B). This, again, suggests a higher sensitivity of human cells to mtDNA-depleting agents.

143B#6 and 5460#5 cells are derivatives of 143B cells in which the TFAM gene was knocked out and rescued with retrovirally encoded wt TFAM cDNA [1]. Like parental 143B cells, these cell lines responded well to 200 μM ddC, which reduced their mtCN to the threshold level in 11–12 days (Figure 2C).

ddC at 200 μM also worked well in other cell lines of human origin (HeLa, A549, HT1080, and HepG2), reducing their mtCN down to the threshold in 10–39 days (Figure 2D). Curiously, mtCN in HT1080 cells dropped rapidly to 1 and then stayed there for an extended period. We established that this phenomenon resulted from the presence in the nuclear genome of these cells of a mitochondrial pseudogene (NUMT) as mtCN was below 1 when using an alternative mtDNA probe.

We tested the efficiency of a related dideoxy compound, stavudine (d4T, 2′,3′-Didehydro-3′-deoxythymidine). This compound failed to affect mtCN in 143B cells at all tested concentrations, which starkly contrasts previous reports [36,41,48] (Figure 2E).

Finally, to validate the utility of the ddC treatment for the isolation of ρ^0^ cell lines, we cloned some cell lines after ddC treatment and analyzed their mtDNA content by duplex PCR. In every attempted case, ρ^0^ clones were isolated (Figure 2F).

### 3.3. A Combination of EtBr and ddC Is Effective for mtDNA Elimination from Mouse/Human Somatic Cell Hybrids

**Figure 3.**
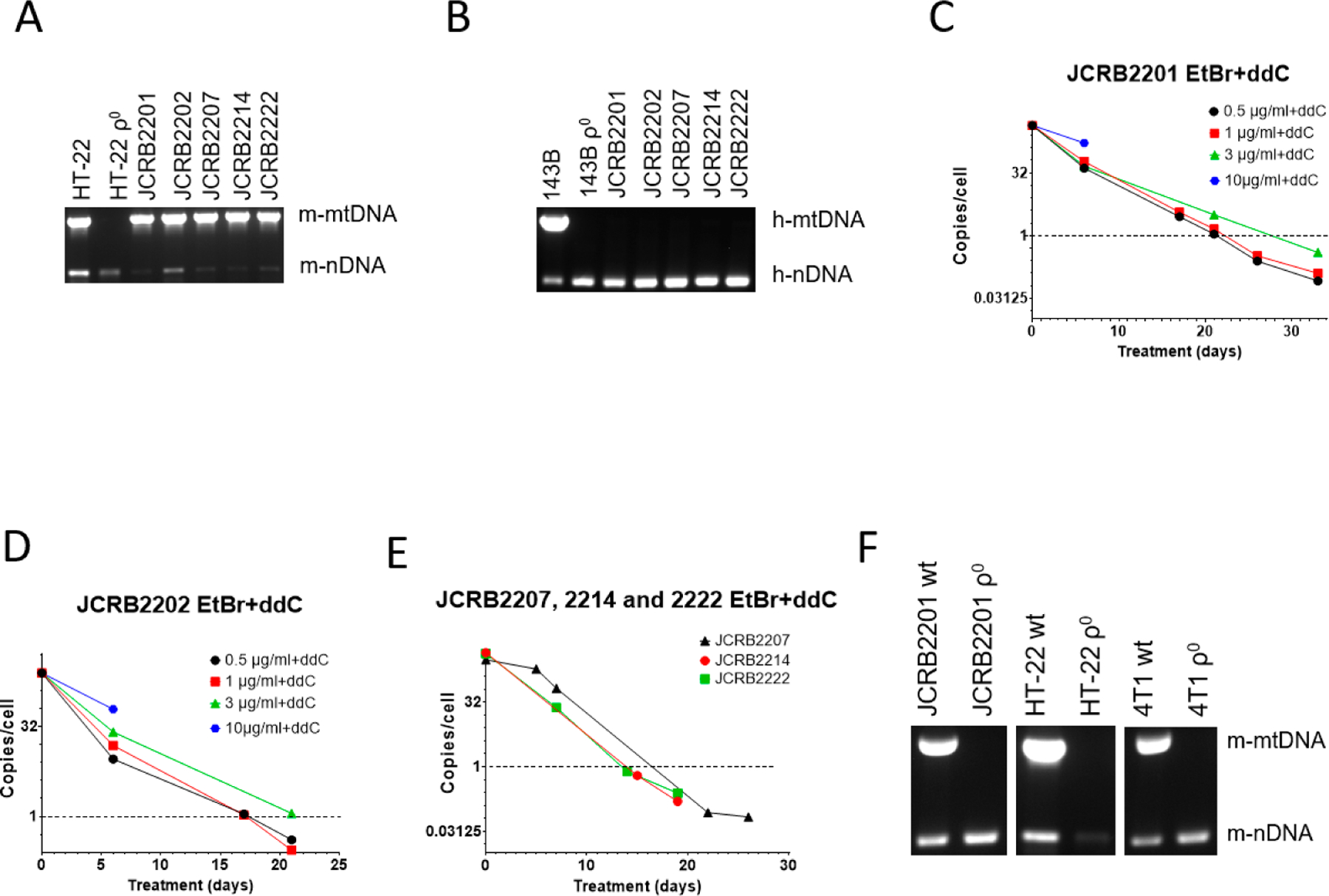
mtDNA depletion in JCRB mouse/human somatic cell hybrids with a combination of EtBr and ddC. A and B, Analysis of JCRB clones for the presence of mouse (**A**) and human (**B**) mtDNA. These hybrids only retain mouse mtDNA. (**C**,**D**) JCRB2201 and JCRB2202 responded similarly to 200 μM ddC plus various concentrations of EtBr in that 10 μg/mL EtBr was cytostatic, 3 μg/mL EtBr had intermediate efficiency, presumably due to residual cytostatic effects, and 0.5 and 1 μg/mL EtBr were similarly effective in mtDNA depletion. (**E**) A combination of 200 μM ddC and 0.5 μg/mL EtBr is effective in mtDNA depletion in JCRB2207, JCRB2214, and JCRB2222 cell lines. (**F**) mtDNA analysis in clones of JCRB2201, HT-22, and 4T1 cells isolated after treatment with 0.5 μg/mL EtBr plus 200 μM ddC. Prior to analysis, clones were expanded in media without either EtBr or ddC.

We were also interested in mtDNA elimination from JCRB human/mouse somatic cell hybrids. These hybrids each contain a single human chromosome and retain mouse but not human mtDNA (Figure 3A,B). This is consistent with previous observations that mouse/human somatic cell hybrids rapidly segregate mtDNA and retain mtDNA from a single species, most commonly mouse [49,50]. In our preliminary studies, we established that mouse/human somatic cell hybrids JCRB2201 and JCRB2202 were resistant to mtDNA depletion with EtBr. Therefore, we decided to test the utility of the combined treatment with 200 μM ddC and a range of EtBr concentrations, which has shown great promise in MEF#5 cells (Figure 1C), for mtDNA elimination from these hybrids.

Similar to MEF#5 and 143B cells, a combination of ddC with a high concentration of EtBr (10 μg/mL) was cytostatic (Figure 3C,D). At 3 μg/mL EtBr+ddC, mtDNA depletion in both cell lines was delayed compared to lower EtBr concentrations (0.5 and 1 μg/mL). This again suggests the negative effects of EtBr intercalation into nuclear DNA, leading to slower proliferation since these effects are also observed without ddC (e.g., Figure 1A).

As a result, we chose the lowest EtBr concentration (0.5 μg/mL) in combination with 200 μM ddC for mtDNA elimination in the remaining JCRB cell lines (JCRB2207, 2214, and 2222). The treatment was effective in all three cell lines (Figure 3E). We also subjected mouse mammary carcinoma 4T1 cells and the HT-22 immortalized mouse hippocampal neuronal cell line to the combined treatment with EtBr and ddC, which resulted in the successful isolation of ρ^0^ clones in both cases (Figure 3F).

## 4. Discussion

With the ever-growing interest in mtDNA biology, the interest in cells devoid of mtDNA also grows. However, their supply remains limited. The methods for generating ρ^0^ cells are time-consuming, and some techniques only apply to a limited subset of cell lines. Therefore, there is continuing interest in developing new techniques for inducing mtDNA loss in cultured cells.

Here, we examined the suitability of zddC/zalcitabine for generating ρ^0^ cells. While in patients zalcitabine only induces moderate mtDNA depletion [36], in our study, the application of this drug to many human and mouse cells in culture resulted in a gradual loss of mtDNA, which enabled the isolation of ρ^0^ derivatives of popular human cell lines, HeLa, A549, 143B, and HT1080, as well as spontaneously immortalized mouse embryonic fibroblasts and single human chromosome-containing mouse/human somatic cell hybrid cell line JCRB2201. We did not pursue the isolation of ρ^0^ derivatives of other JCRB cell lines because of the lack of interest or availability of the ρ^0^ derivatives obtained by other techniques. In contrast, another antiretroviral drug with a similar mechanism of action, stavudine, did not induce mtDNA depletion in 143B cells, although it does cause mtDNA depletion in patients [36], experimental animals [41], and cultured mesenchymal stem cells [48].

Our study and previous experience allowed us to make several generalizations. First, EtBr still remains an effective means of inducing the ρ^0^ state, although the susceptibility of cell lines to this drug varies dramatically. At 500 ng/mL (~1.27 μM), EtBr blocked proliferation and induced cell death in 143B cells, whereas this concentration was too low to induce complete loss of mtDNA in MEFs and resulted in the development of resistance. This observation highlights the need to carefully calibrate the EtBr concentration for each cell line, which is undesirable from a practical standpoint. Moreover, EtBr is mutagenic [51,52], and treatment with this compound induces nuclear DNA (nDNA) rearrangements [53]. Also, EtBr affects nuclear transcription [54] and can block cell proliferation, as shown in this study.

Second, unlike EtBr, ddC was tolerated well by all cell lines tested in this study at 200 μM, the highest concentration used.

Third, cell lines of murine origin tend to be more resistant to the action of mtDNA-depleting agents (in our study, both EtBr and ddC). This may result from the reduced permeability of the plasma membrane, mitochondria, nucleoids, the higher tolerance of mtDNA replication apparatus, or any combination of these factors. However, it is remarkable that the trend is the same in two chemically distinct drugs that act by different mechanisms.

Fourth, the combination of lower EtBr concentrations with ddC prevents the cytostatic effects of EtBr on cellular proliferation while achieving the goal of mtDNA elimination. This is important for two reasons. First, active proliferation helps to achieve mtDNA depletion through dilution in actively proliferating cells upon the blockage of mtDNA replication. Second, nonproliferating cells eventually die.

## 5. Conclusions

In conclusion, we believe that ddC will become a useful addition to the arsenal of tools for inducing the ρ^0^ state in cultured cells.

## Figures and Tables

**Table 1.** Oligonucleotides used in this study.

Purpose	Name	Sequence	Amplicon, bp
Genotyping of the ρ^0^ state in human cells	hMitF	AATGTCTGCACAGCCACTTTCCAC	901
	hMitR	TCGTAGTGTTCTGGCGAGCAGTTT	
	hNucF	CGGACAGGATTGACAGATTGA	389
	hNucR	AGCTTATGACCCGCACTTAC	
Genotyping the ρ^0^ state in murine cells	mMitF	AAAGCATCTGGCCTACACCCAGAA	1041
	mMitR	ACCCTCGTTTAGCCGTTCATGCTA	
	mNucF	CCACGTGCTCTGTATGAGATT	636
	mNucR	ATGCTGGCTTATCTGTTCCTT	
mtDNA copy number determination in human cells by dddPCR	NucF	AACTTGTAAGTGGTAGTGCATAGA	N/A
	NucR	GTAGGAGGACATTTGAGGAGTG	
	NucProbe	FAM-TCAGGCAGACTGACACTAGAGTTCACA-BHQ1	
	MitF	CTGATCAGGGTGAGCATCAAA	
	MitR	GAATGATGGCTAGGGTGACTTC	
	MitProbe	Hex-TGCGAGCAGTAGCCCAAACAATCT-BHQ1	
mtDNA copy number determination in mouse cells by dddPCR	NucF	CCTGGGCTTTGAACTTGTCTA	N/A
	NucR	TGAGGGCATTGGAGATTGTG	
	NucProbe	FAM-TGGTCCTGCTATCAGAGATGCAACG-BHQ1	
	MitF	GGCCTATTAATCGCAGCTACAG	
	MitR	GTAGTGCTGAAACTGGTGTAGG	
	MitProbe	FAM-ATTTGGCCTCCACCCATGACTACC-BHQ1	

## Data Availability

The data presented in this study are contained in the article.
